# miR-3529-3p/ABCA1 axis regulates smooth muscle cell homeostasis by enhancing inflammation via JAK2/STAT3 pathway

**DOI:** 10.3389/fcvm.2024.1441123

**Published:** 2024-08-27

**Authors:** Tingyu Wang, You Yu, Yinglong Ding, Ziying Yang, Shumin Jiang, Faxiong Gao, Shan Liu, Lianbo Shao, Zhenya Shen

**Affiliations:** Department of Cardiovascular Surgery of the First Affiliated Hospital of Soochow University & Institute for Cardiovascular Science, Soochow University, Suzhou, China

**Keywords:** thoracic aortic dissection, smooth muscle cell, homeostasis, miR-3529-3p, ABCA1

## Abstract

**Background:**

Thoracic Aortic Dissection (TAD) is a life-threatening disease without effective drug treatments. The disruption of HASMCs homeostasis is one direct histopathologic alteration in TAD pathological process. Several miRNAs have been shown abnormally expressed in TAD and to regulate HASMCs homeostasis. The primary goal of this study is to identify the miRNAs and the specific mechanisms that lead to HASMCs homeostasis disruption.

**Methods:**

Bulk miRNA sequencing was performed to explore the aberrantly expressed miRNA profile in TAD, and differentially expressed miRNAs were verified with qRT-PCR. To explore the role of the key miRNAs (miR-3529) in HASMCs homeostasis, we overexpressed this miRNA with lentivirus in HASMCs. Integrative transcriptomics and metabolomics analysis were used to uncover the functional roles of this miRNA in regulating HASMCs homeostasis. Further, the target gene of miR-3529 was predicted by bioinformatics and verified through a dual-luciferase reporter assay.

**Results:**

Bulk miRNA sequencing showed miR-3529 was elevated in TAD tissues and confirmed by qRT-PCR. Further experimental assay revealed miR-3529 upregulation induced HASMCs homeostasis disruption, accompanied by reducing contractile markers and increasing pro-inflammatory cytokines. Integrative transcriptomics and metabolomics analysis showed that miR-3529 overexpression altered the metabolic profile of HASMC, particularly lipid metabolism. ABCA1 was found to be a direct target of miR-3529. Mechanistically, the miR-3529/ABCA1 axis disrupted HASMCs homeostasis through the JAK2/STAT3 signaling pathway.

**Conclusions:**

miR-3529 is elevated in TAD patients and disrupts HASMCs homeostasis by reprogramming metabolism through the JAK2/STAT3 signaling pathway. These findings favor a role for miR-3529 as a novel target for TAD therapy.

## Graphical Abstract

Modulation of the miR-3529/ABCA1/JAK2/STAT3 signaling pathway and its functional impact on disruption of HASMCs homeostasis associated with TAD.

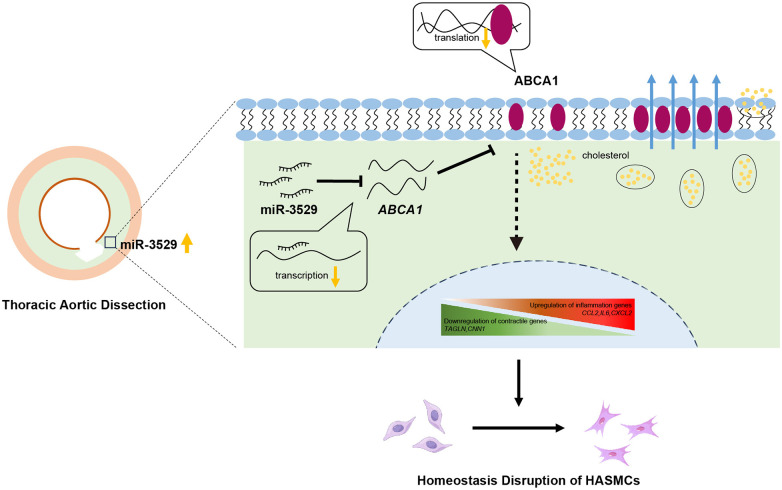

## 1 Introduction

Thoracic aortic dissection (TAD) is one serious acute cardiovascular disease with high mortality. It is characterized by the separation of aortic wall layers by extraluminal blood entering the vessel wall from an intimal break (1, 2). Open surgery and endovascular repair remain the major approaches for TAD treatment, however, their long-term prognosis is still poor (3). The pathological mechanisms of TAD are complex and still not fully understood, further investigations are necessary to develop potential targets for noninvasive TAD therapy.

Vascular smooth muscle cells (VSMCs) are the fundamental component of the tunica media of arteries and are essential for maintaining vascular contraction, these highly specialized cells possess variable phenotypes. In healthy arteries, VSMCs are in a quiescent homeostasis state that is characterized by the expression of contractile proteins, such as actin alpha 2 and transgelin (TAGLN) (4–6). A variety of environmental stimuli can induce their homeostasis disruption which presents a proliferative and secretory phenotype (7). In addition, VSMCs also contribute to vascular inflammation by acquiring pro-inflammatory molecular and cellular features (8–10). The homeostasis alteration and phenotype switch of VSMCs is driven by metabolism reprogramming, aggravating the progression of several vascular diseases (11, 12).

It has been described miRNAs are promising novel biomarkers for the diagnosis and treatment targets of cardiovascular disease (13–16). Abnormal expressed miRNAs participate in the development and regulation of aortic dissection by modulating VSMCs phenotype (17–19). For instance, miR-128 directly modulates VSMCs differentiation and function by regulating the DNA methylation level of myosin heavy chain 11 via inhibiting Krüppel-like factor 4 expression (19).

In our present study, we discovered that the miR-3529-3p (miR-3529) level aberrant increased in the aortic media of TAD. Further, we demonstrated that overexpressing miR-3529 induces human aortic smooth muscle cells (HASMCs) homeostasis disruption. Gain- and loss-of-function experiments revealed that this miRNA modulates HASMCs homeostasis by directly targeting ATP-binding cassette subfamily A member 1 (ABCA1) and regulating JAK2/STAT3 signaling pathway activation. It is indicated that miR-3529 might be an effective therapeutic target for the treatment of TAD in clinical in the future.

## 2 Materials and methods

### 2.1 Cell culture

Primary HASMCs were obtained from Yubo Biotechnology Co., LTD, and maintained in DMEM/F12 (Gibco, C11330500BT) supplemented with 10% FBS. HEK-293 T cells were obtained from ATCC and cultured for lentivirus preparation in DMEM/high glucose medium (Gibco, C11960500BT) containing 10% FBS. Cells were cultured in a humidified 5% CO_2_ atmosphere at 37 °C.

### 2.2 Lentivirus preparation and cell transfection

Lentiviral particles were prepared by co-transfection of a plasmid encoding miR-3529 and packing plasmids in HEK-293 T cells. The mature miR-3529 sequence (Supplementary Table S1 for sequence fragment) was cloned into pCDH-CMV-MCS-EF1-CopGFP-T2A-Puro vector (System Biosciences, CD513B-1). Then, HEK-293 T was transfected with the lentiviral plasmid and psPAX2, pMD2.G packaging plasmids. After transfection for 48 h, the supernatant was collected, and viral particles were concentrated. Then, primary HASMCs were infected with lentiviral particles expressing miR-3529 or control lentiviral particles. 48 h after transfection, the positive ratio of the transfected cells was determined, and the miR-3529 expression was measured by qRT-PCR. miR-3529 inhibitor was synthesized and transfected into cells to suppress miR-3529 expression following the manufacturer's protocols.

### 2.3 Induction inflammatory reactions in HASMCs

HASMCs were seeded at 3 × 10^5^ cells/well into a 6-well plates and stimulated with 20 ng/ml IL-1β (Peprotech, 200-01B) in basic DMEM/F12 medium for 24 h.

### 2.4 Cholesterol loading and Oil Red O staining

Cholesterol was loaded to cells by using Chol:MβCD complex (Sigma, C4951) which contains 50 mg cholesterol/g solid. Subconfluent HASMCs were incubated with Chol:MβCD (20 µg/ml) for 48 h, then fixed with 4% paraformaldehyde for 10 min and stained with Oil Red O (Beyotime, C0157S) for 30 min. Cells were examined with an optical microscope (Olympus, Japan), and pictures of representative fields were taken.

### 2.5 Human vascular specimen collection

This study was approved by the Institution Review Board of the First Affiliated Hospital of Soochow University in accordance with the Declaration of Helsinki (Approval Number: 2020-478). Tissues of the aortic aorta were obtained from patients undergoing TAD repair operations. Patients with traumatic aortic injury, inflammatory aortic disease, Ehlers-Danlos syndrome, and Marfan syndrome were excluded.

### 2.6 RNA extraction and qRT-PCR

Total RNA was isolated using RNAiso Plus (Takara, 9108) according to the manufacturer's protocol. For mRNA expression detection, RNA was reversely transcribed with PrimeScript RT reagent Kit (Takara, RR037A), and qRT-PCR was performed using TB Green Premix Ex Taq (Takara, RR420A). The mRNA expression levels were normalized to those of 18s. For miRNA expression, RNA was reversely transcribed using miRNA 1st Strand cDNA Synthesis Kit (Vazyme, MR101-01). miRNA Universal SYBR qPCR Master Mix (Vazyme, MQ101-02) was used to perform qRT-PCR for mature miRNAs, and U6 was used for an internal control. Primers used in this study are listed in Supplementary Table S2.

### 2.7 Western blot analysis

HASMCs were lysed in RIPA lysis Buffer (Beyotime, P0013C) with PMSF. Proteins were separated by SDS-PAGE and subsequently transferred onto PVDF membranes (Millipore, ISEQ00010). The membranes were blocked with 5% BSA in TBST and then incubated with primary antibodies at 4 °C overnight. Following incubation with HRP-conjugated secondary antibodies (Cell Signaling Technology, 7074P2) for 1 h at room temperature, the bands were analyzed using the ChemiDoc XRS imaging system (Bio-Rad, USA). Primary antibodies used in this study are listed in Supplementary Table S3.

### 2.8 EdU incorporation assay

EdU (5-ethynyl-2'-deoxyuridine) incorporation assays were performed using a BeyoClick EdU Cell Proliferation Kit (Beyotime, C0075S) following the manufacturer's instructions. EdU-positive nuclei in HASMCs were captured by fluorescence microscope (Olympus, Japan), and quantification of the percentage of EdU^+^ cells was performed with ImageJ software.

### 2.9 H&E staining, EVG staining and immunofluorescence staining

Tissue samples were embedded in paraffin post-fixed with paraformaldehyde. Next, paraffin-embedded tissues were cut into 5 µm-thick slices and performed H&E Staining (Solarbio, G1120) and Verhoeff-van Gieson (EVG) staining (Solarbio, G1597). For immunofluorescence staining, tissues or cells were permeabilized with 0.1% Triton X-100 and blocked with 5% BSA, followed by incubation with TAGLN (Proteintech, 60213-1-Ig), ABCA1 (Proteintech, 26564-1-AP) primary antibodies at 4°C overnight. Following 1 h of incubation with fluorescently labeled secondary antibodies at room temperature, the sections were observed and photographed by fluorescence microscope (Olympus, Japan) or laser confocal microscope (Zeiss, Germany).

### 2.10 Transcriptome and metabolome sequencing and integrated analysis

Transcriptome and metabolome sequencing were performed by BGI. For transcriptome analysis, differentially expressed genes (DEGs) analysis was performed using the DESeq2 package with Q value <0.05 and fold change (FC) >1.5. To explore the underlying biological functions and pathways, Gene Ontology (GO) and Kyoto Encyclopedia of Genes and Genomes (KEGG) enrichment analysis of DEGs was conducted using the “phyper” function within the R project.

Metabolome sequencing was measured using an LC-MS/MS system. The annotations of the detected accurate metabolites were queried and aligned to BGI Metabolome Database (BMDB), mzCloud database, and ChemSpider database. The differentially expressed metabolites (DEMs) were determined based on the combination of a statistically significant threshold of variable importance in projection >1.0, Student's *t*-test *p* < 0.05, and FC > 1.2.

The bioinformatic analysis entailed the discernment of notably perturbed mRNA and metabolite profiles utilizing a blend of multi-dimensional and single-dimensional analytical methodologies. Spearman correlation analysis was used to unveil the associations between DEGs and DEMs, thus providing crucial insights into understanding the complex interaction of metabolites and genes that mutually influence biological processes. Canonical Correlation Analysis (CCA) was performed to identify the correlation and covariation between the co-regulated metabolites and genes.

### 2.11 Luciferase reporter assay

To confirm whether *ABCA1* is a target gene of miR-3529, the segment of *ABCA1* 3'UTR containing miR-3529 binding site (5'-ATGCATATTTCTATGTTGTAA-3’, the seeded sequence was underscored) and a segment of mutant-type 3'UTR were cloned into a psiCHECK-2 Luciferase Vector (Promega, C8021), respectively. The recombinant plasmid (either wild or mutant type) and miR-3529 mimic were co-transfected into the HEK-293 *T* cells. After 24 h, the luciferase activity was measured by the Firefly & Renilla Luciferase Reporter Assay Kit (Meilunbio, MA0518-1) according to the manufacturer's protocol.

### 2.12 Regulation of ABCA1 expression

Subconfluent HASMCs were treated with T0901317 (a liver X receptor agonist, MedChemExpress, HY-10626) for 12 h to stimulate ABCA1 expression as previous reported (20). The HASMCs treated solely with DMSO were employed as the control group.

### 2.13 Statistical analysis

Statistical differences were evaluated by Student's *t*-test (two groups) or ANOVA. All the statistical tests were two-tailed. Analysis of the data and plotting of the figures were performed by using GraphPad Prism 9.0 software (GraphPad Software, USA). A *p*-value less than 0.05 is considered statistically significant in all experiments.

## 3 Results

### 3.1 Bioinformatic analysis of miRNAs profiles and validation of the dysregulated miRNAs in TAD

**Figure 1:**
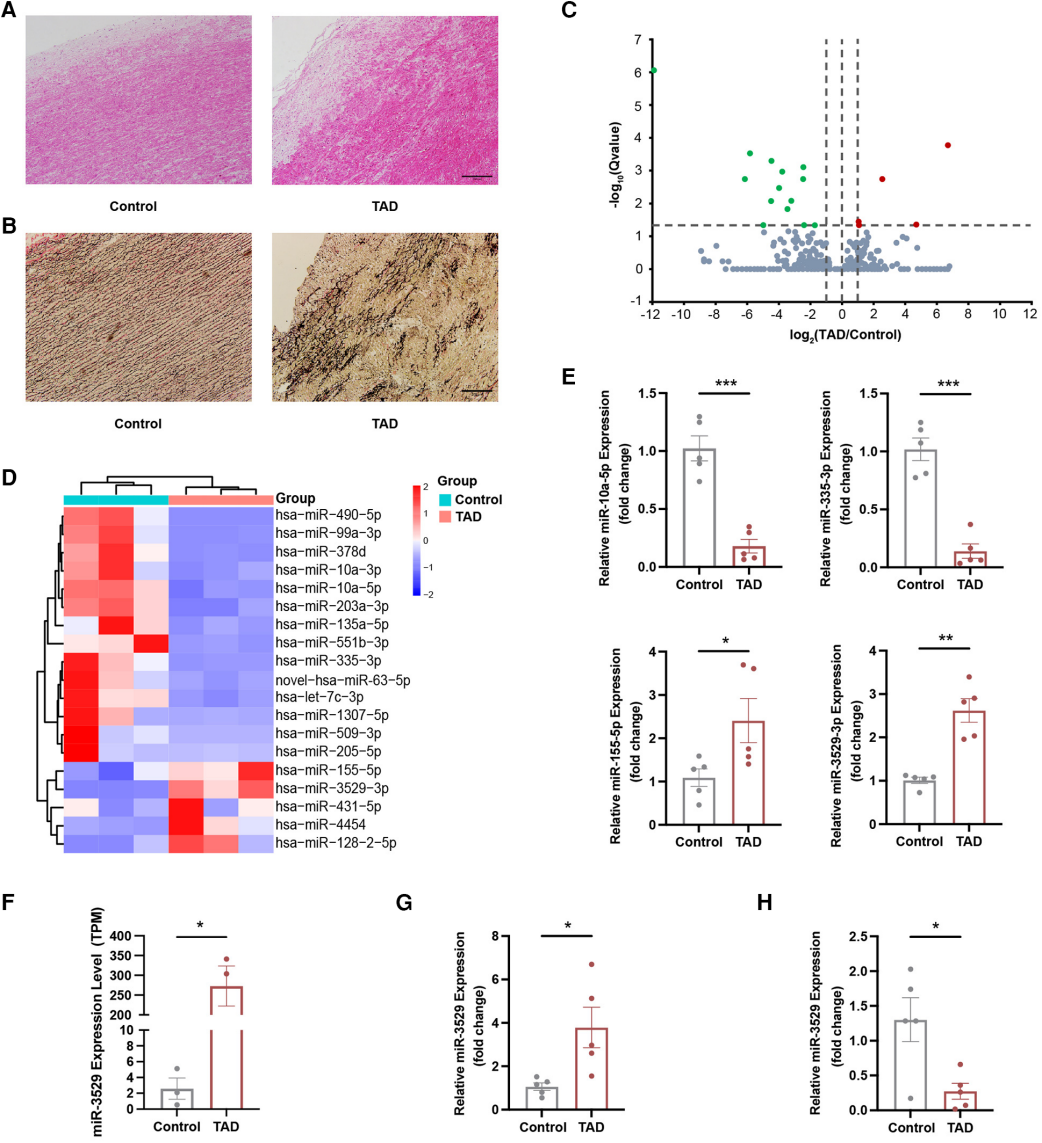
miR-3529 expression is upregulated in TAD tissues. **(A,B)** H&E staining and EVG staining of TAD and control tissues, scale bar, 200 µm. **(C)** Volcano plot, with green dots representing significantly downregulated miRNAs and red dots representing significantly upregulated miRNAs in TAD and control tissues. **(D)** Hierarchical clustering heat map of 19 miRNAs differentially expressed between control and TAD tissues. **(E)** qRT-PCR verification of 4 candidate miRNAs for microarray data in TAD and control tissues (*n* = 5). **(F)** The expression level (TPM) of miR-3529-3p detected by miRNA sequencing (*n* = 3). **(G)** qRT-PCR detection of miR-3529 in tunica media of TAD and control tissues (*n* = 5). **(H)** qRT-PCR detection of miR-3529 in tunica intima of TAD and control tissues (*n* = 5). Data are presented as means ± SEM. *P < 0.05, **P < 0.01, ***P < 0.001.

Aortic injury was evaluated by H&E staining and EVG staining as shown in Figures 1A,B. Compared to normal aorta, the dissected section exhibits irregularities and layered tearing of the vascular wall (Figure 1A). EVG staining exhibited more obvious aortic wall damage with elastin fragmentation or breakage of TAD patients (Figure 1B). miRNA sequencing was performed on vascular tissues from TAD patients and control. There were 380 miRNAs identified, among them 19 miRNAs were differentially expressed, including 5 upregulated miRNAs and 14 decreased ones (Figure 1C). Furthermore, heatmaps of these differentially expressed miRNAs were used in hierarchy cluster analysis suggested they can be distinguished between aortic dissection group and control group (Figure 1D, and the TPM value were listed in Supplementary Table S4). Four aberrantly expressed miRNAs (two upregulated ones—miR-155-5p, miR-3529-3p, and two downregulated ones—miR-10a-5p, miR-335-3p) were selected for validation with qRT-PCR analyses. The results showed miR-10a-5p and miR-335-3p were deceased, and miR-155-5p and miR-3529-3p were increased in the aorta of TAD (Figure 1E), which were generally consistent with those of miRNA sequencing.

The bulk miRNA sequencing showed that miR-3529-3p (miR-3529) is hardly expressed in control group, and presents abundant expression in the TAD tissues (Figure 1F). To determine the localization of miR-3529 in the layer of aotia, we evaluated its expression in tunica and intima respectively. The results showed miR-3529 was upregulated in the tunica media and downregulated in the intima of TAD (Figures 1G,H), which suggested it is mainly elevated in the tunica media of the diseased aorta. Therefore, we speculated that miR-3529 may disrupt VSMCs homeostasis and lead to media degradation in TAD.

### 3.2 miR-3529 overexpression disrupts HASMCs homeostasis

**Figure 2:**
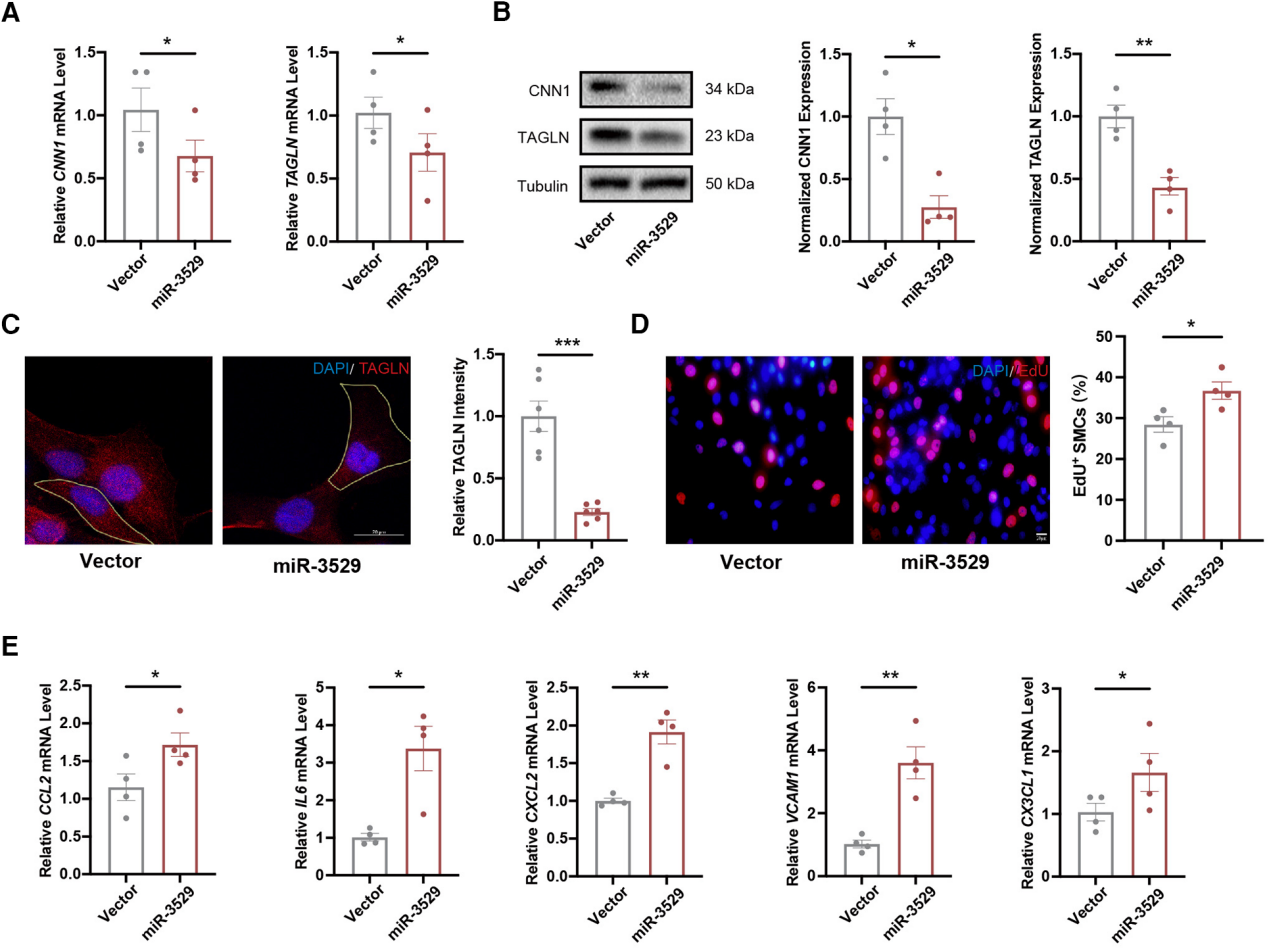
miR-3529 disrupts HASMCs homeostasis *in vitro*. **(A)** Expression of HASMCs contractile markers (*CNN1* and *TAGLN*) in miR-3529-overexpressing HASMCs vs. CTR cells, measured by qRT-PCR (*n* = 4). **(B)** Representative Western blots and quantification of HASMCs contractile marker proteins (CNN1 and TAGLN) expression in miR-3529-overexpressing HASMCs vs. CTR cells (*n* = 4). **(C)** Staining of miR-3529-overexpressing HASMCs vs. CTR cells for TAGLN (red) (representative images chosen for similarity to the global quantification; scale bar, 20 µm; *n* = 6). **(D)** Representative images of EdU staining (red) and quantification of proliferative HASMCs, scale bar, 20 μm. **(E)** Expression of HASMCs pro-inflammatory genes (CCL2, IL6, CXCL2, VCAM1, and CX3CL1) in miR-3529-overexpressing HASMCs vs. CTR cells treated with IL-1β (20 ng/ml, 24 h), measured by qRT-PCR (*n* = 4). Data are presented as means ± SEM. *P < 0.05, **P < 0.01, ***P < 0.001.

To explore the role of miR-3529 in regulating HASMCs homeostasis, miR-3529 was overexpressed by lentivirus transfection (Supplementary Figures S1A,B). The expression of contractile markers calponin 1 (CNN1) and TAGLN have significantly reduced both at RNA and protein levels (Figures 2A,B). miR-3529 induces instability and subsequent reorganization of the intracellular cytoskeleton, as demonstrated by the decrease of TAGLN (Figure 2C). This structural change was associated with an increased cell size (Supplementary Figure S2A). These strongly suggest that this miRNA regulates the homeostasis status of VSMCs. Analysis of VSMCs homeostasis-associated phenotypes, including cell proliferation and proinflammatory genes supported this notion (21, 22). EdU staining showed that miR-3529 promotes the proliferation of HASMCs (Figure 2D). Several inflammatory cytokines including IL6, CCL2, CXCL2, VCAM1, and CX3CL1 were also detected with miR-3529 overexpression, and the results showed miR-3529 did not alter their expression obviously in normal condition (Supplementary Figure S3). Interestingly, when exposed to an inflammatory stimulus, these cytokines exhibited a marked upregulation in HASMCs as expected (Supplementary Figure S4). Further, the proinflammatory effect was more pronounced with miR-3529 overexpression in normal (Figure 2E). However, by inhibiting miR-3529 expression with specific inhibitors in primary HASMCs (Supplementary Figure S5A), these mentioned homeostasis-related phenotypes that induced by miR-3529 were not altered (Supplementary Figures S5B,D). This may be due to its low background expression levels (Supplementary Tables S5, S6). Overall, these findings confirmed the role of miR-3529 in regulating HASMCs homeostasis.

### 3.3 Normalization of miR-3529 level rescues HASMCs homeostasis disruption

**Figure 3:**
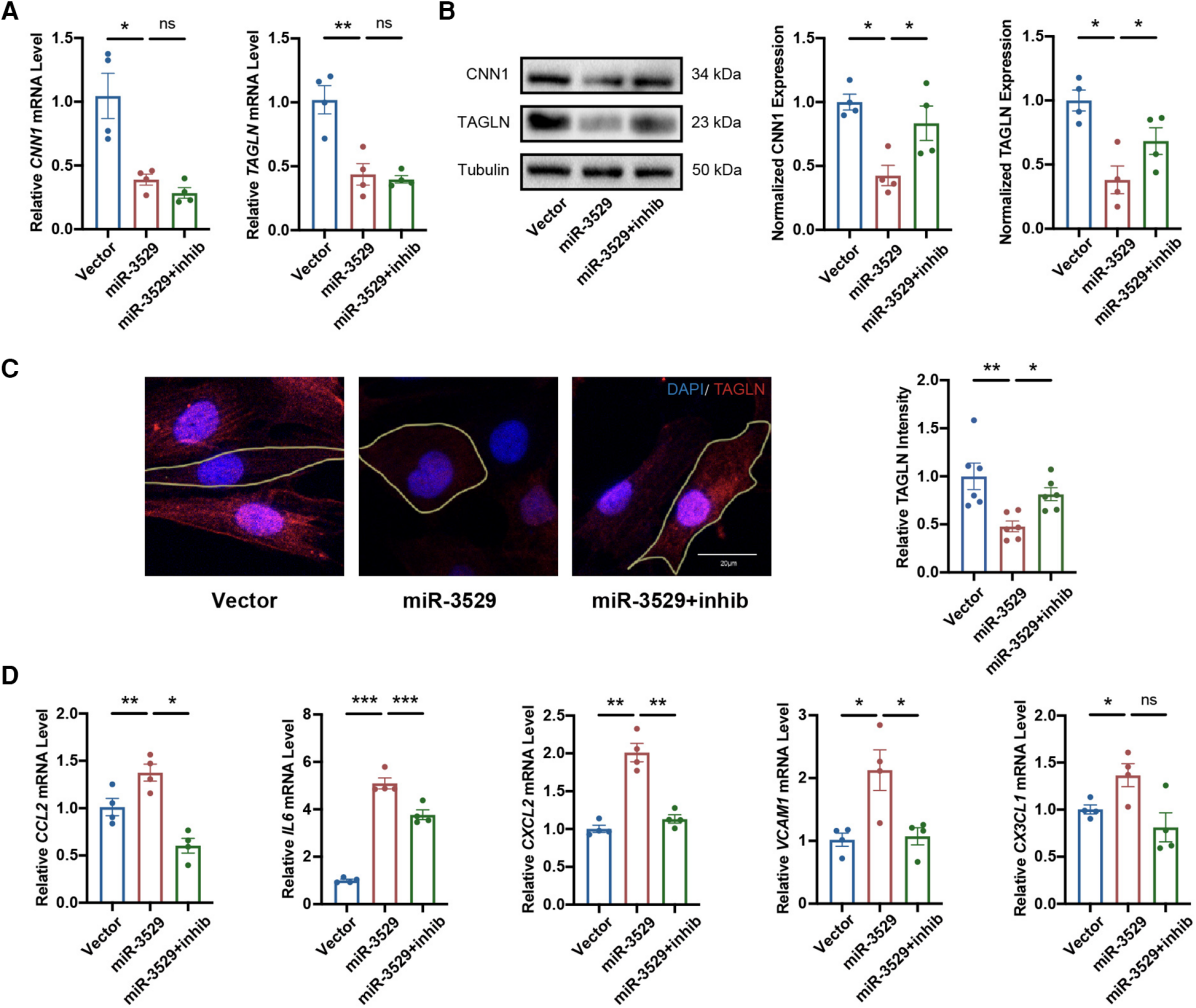
Normalization of miR-3529 restores the feature of HASMCs homeostasis. **(A)** Expression of HASMCs contractile markers in miR-3529-overexpressing HASMCs transfected with miR-3529 inhibitor (miR-3529 + inhib) or CTR inhibitor (miR-3529) compared to CTR cells transfected with CTR inhibitor (Vector), measured by qRT-PCR (*n* = 4). **(B)** Representative Western blots and quantification of HASMCs contractile marker proteins expression (*n* = 4). **(C)** Staining for TAGLN (red) (representative images chosen for similarity to the global quantification; scale bar, 20 µm; *n* = 6). **(D)** qRT-PCR analysis of HASMCs pro-inflammatory genes in miR-3529 differently expressed HASMCs treated with IL-1β (*n* = 4). Data are presented as means ± SEM. *P < 0.05, **P < 0.01, ***P < 0.001, ns, not significant.

To strengthen the evidence of miR-3529 being able to regulate HASMCs homeostasis, we performed “rescue” experiments on miR-3529-overexpressing HASMCs by transducing a specific inhibitor (Supplementary Figure S1C). With miR-3529 normalization, HASMCs function of the contractile phenotype was enhanced (Figures 3A,B). Meanwhile, stress fiber formation, cell size, and inflammation were “rescued” (Figures 3C,D; Supplementary Figure S2B). These results indicate that miR-3529 upregulation in HASMCs has a profound role in modulating HASMCs homeostasis.

### 3.4 Identification of differentially expressed genes (DEGs) altered by miR-3529 and functional annotation

**Figure 4:**
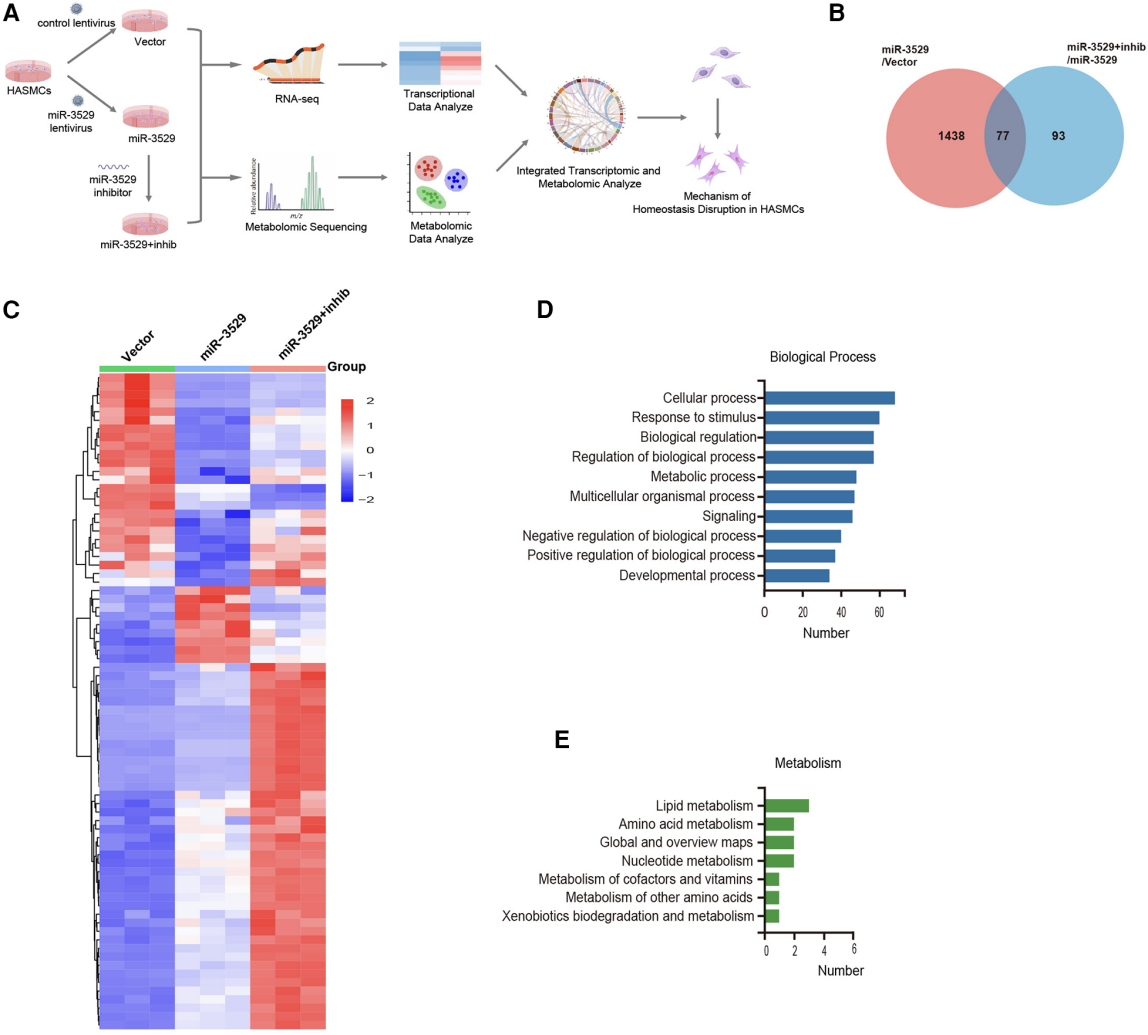
General analysis of transcriptome sequences in miR-3529 differently expressed HASMCs. **(A)** Integrated metabolic-transcriptional profiling and analysis pipeline in miR-3529 differently expressed HASMCs. **(B)** Venn diagram showing the overlapping DEGs in HASMCs with different miR-3529 expression. **(C)** Hierarchical clustering heat map of the above 77 DEGs in miR-3529 differently expressed HASMCs. **(D)** GO analysis (biological process) of the above 77 DEGs. **(E)** KEGG pathway analysis (metabolism) of the above 77 DEGs.

To address the mechanism of miR-3529 on HASMCs homeostasis, an integrative transcriptomics and metabolomics analysis was performed in miR-3529 differently expressed HASMCs (Vector group; miR-3529 group; miR-3529 + inhib group) (Figure 4A). From transcriptomics sequencing, there were 77 DEGs identified both in miR-3529 group vs. Vector group set and miR-3529 + inhib group vs. miR-3529 group set (Figure 4B). The clustered heatmap illustrates the differential expression patterns of these 77 genes across the three groups (Figure 4C). Further, GO and KEGG pathway analyses were also conducted to gain insight into molecular functions regulated by miR-3529 in HASMCs. Biological process of GO enrichment analysis showed most pathways upon miR-3529 regulation were involved in response to stimulus and metabolic process (Figure 4D). Metabolism of KEGG analysis further identified lipid metabolism as the most enrichment pathway (Figure 4E). Taken together, these results demonstrate that miR-3529 is a novel regulator of lipid metabolism in HASMCs.

### 3.5 miR-3529 alters the metabolic expression profile of HASMCs

**Figure 5:**
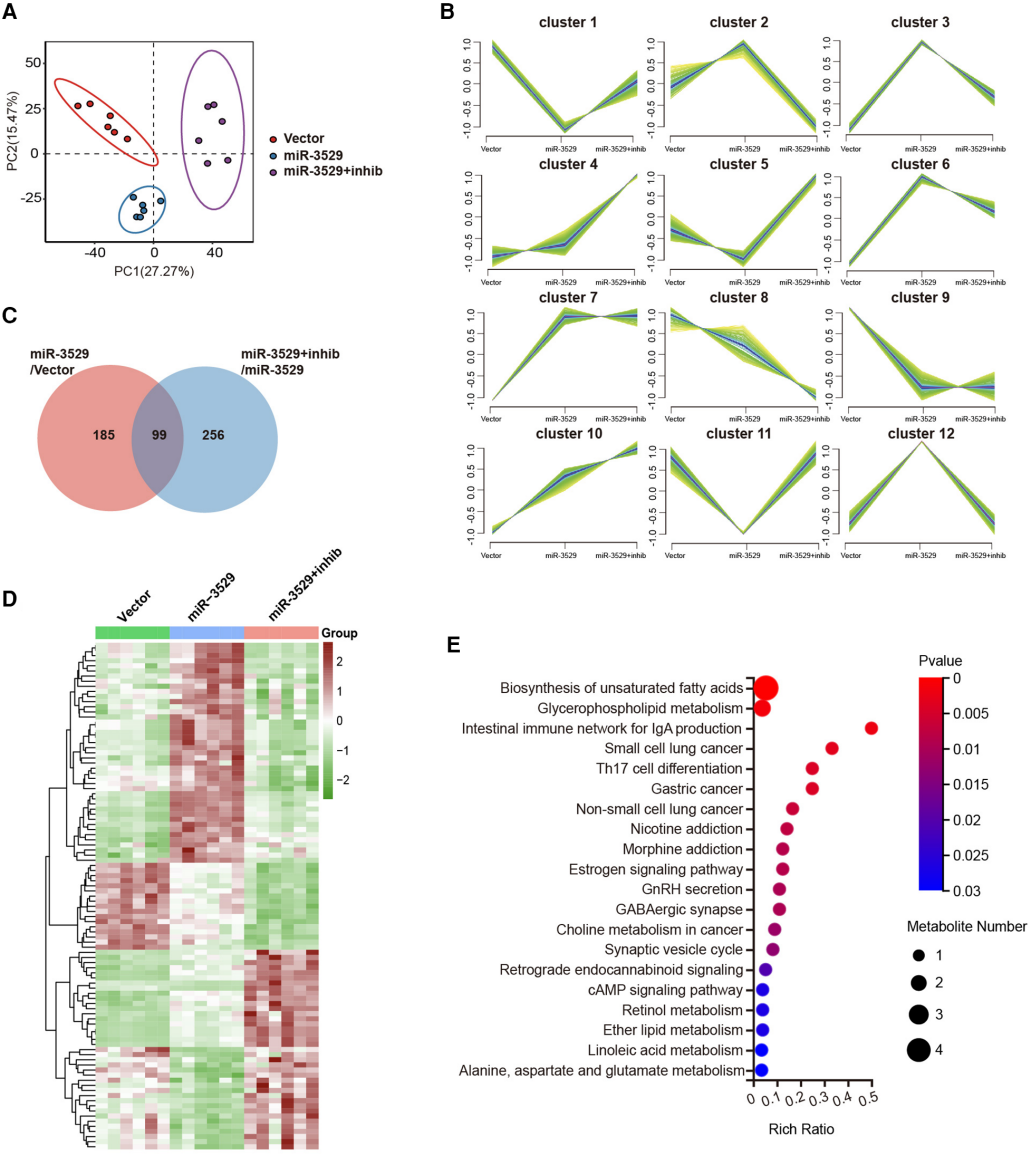
The metabolic expression profile alteration in miR-3529 differently expressed HASMCs. **(A)** Score plot of the partial least squares discriminant analysis (PLS-DA) based on LC/MS data. **(B)** Hierarchical clustering performed on the metabolite expression profiles. All metabolites were classified into 12 major clusters. **(C)** Venn diagram showing the overlapping DEMs in HASMCs with different miR-3529 expression. **(D)** Hierarchical clustering heat map of the above 99 DEMs in miR-3529 differently expressed HASMCs. **(E)** KEGG pathway analysis of the above 99 DEMs**.**

Metabolomics was performed to reveal the metabolic characteristics of HASMCs regulated by miR-3529, there were 1,617 metabolites screened and identified. Partial Least Squares Discriminant Analysis (PLS-DA) was performed to visualize the separations between experimental groups. During the expression of miR-3529, the PLS-DA result declared an obvious separation, while the duplicate samples were compactly clustered together (Figure 5A). The metabolite ion intensity data were centralized and standardized to perform clustering analysis. The metabolites were divided into 12 clusters through the expression pattern between groups (Figure 5B). Each group was carried out to further analyze the DEMs and their associated biological processes regulation network. There were 99 DEMs identified both in miR-3529 group vs. Vector group and miR-3529 + inhib group vs. miR-3529 group, and the expression pattern is screened as shown in the heatmap with clustering analysis (Figures 5C,D). KEGG functional enrichment analysis revealed that most DEMs were enriched in biosynthesis of unsaturated fatty acid and glycerophospholipid metabolism (Figure 5E). This further confirmed the important role of miR-3529 in regulating lipid metabolism.

### 3.6 Integrated analysis of the transcriptome and metabolome

**Figure 6:**
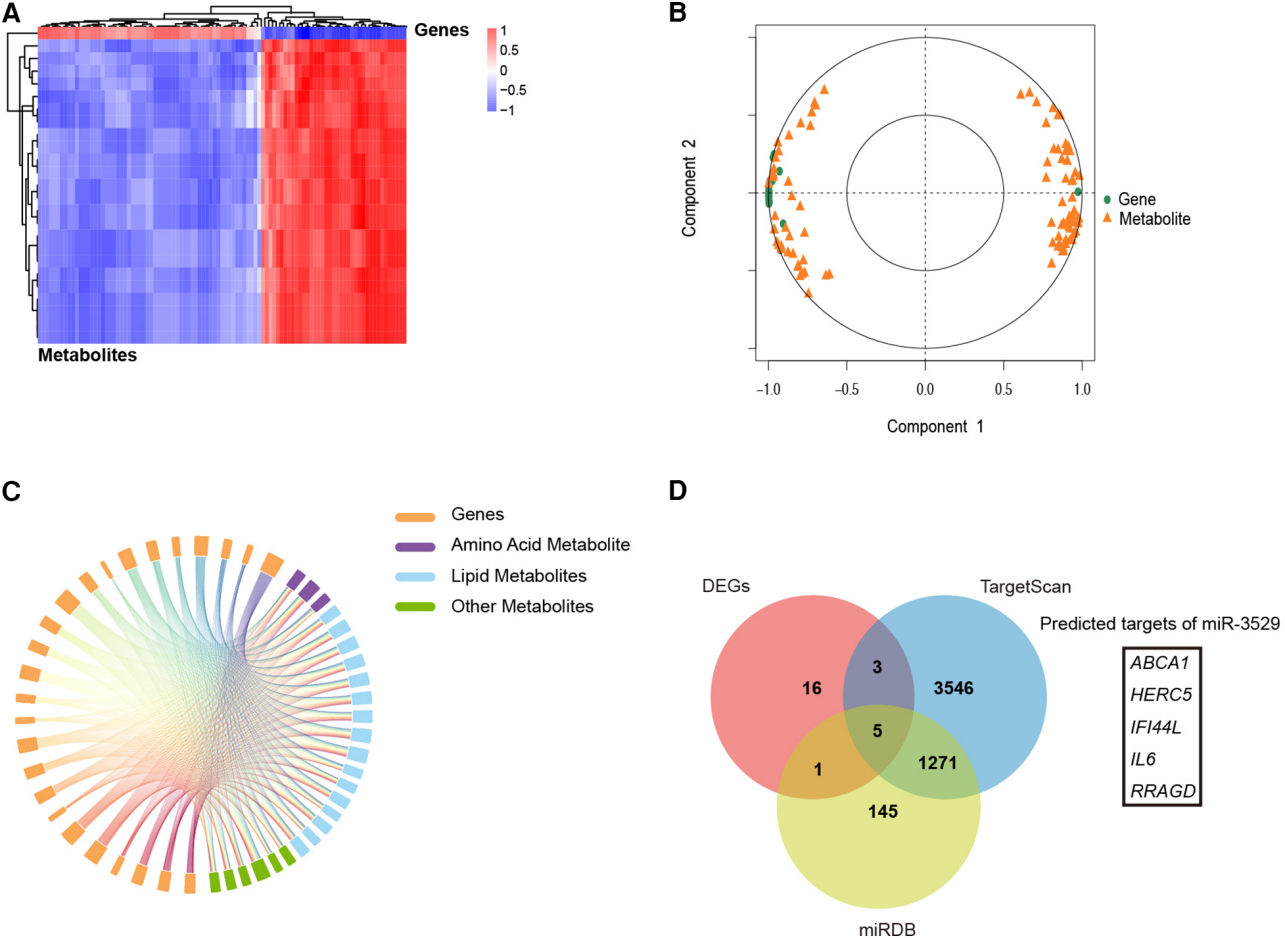
Integrated analysis of the transcriptome and metabolome. **(A)** Spearman correlation heatmap of 77 DEGs with 99 DEMs in miR-3529 differently expressed HASMCs. **(B)** CCA of 77 DEGs with 99 DEMs. **(C)** A correlation chord diagram analysis of DEGs and DEMs. **(D)** 5 DEGs out of 25 DEGs (shown in the above chord diagram) were predicted to be downstream targets of miR-3529.

Multi-omics integrated approach between the screened DEMs and DEGs was employed. Spearman correlation analysis revealed that a total of 25 DEMs were significantly associated with 77 DEGs (Figure 6A). Canonical Correlation Analysis (CCA) reflected the overall correlation between two sets of DEGs and DEMs. It can be seen that there is a significant positive or negative correlation between these DEGs and DEMs (Figure 6B). A correlation chord diagram was performed on the top 25 genes and metabolites with the strongest associations and showed that these metabolites were simultaneously regulated by multiple genes, most of which are lipid substances, suggesting miR-3529 overexpression provides an abnormal state of lipid metabolism (Figure 6C). Finally, the putative targets of miR-3529 were predicted by algorithms (TargetScan and miRDB) and intersected with the mentioned 25 selected genes (23, 24). 5 potential targets were obtained, in which, *ABCA1* is the only one that directly regulating lipid metabolism (Figure 6D). Therefore, *ABCA1* is selected as the putative target of miR-3529 for further validation.

### 3.7 *ABCA1* is a novel direct target of miR-3529 in HASMCs

**Figure 7:**
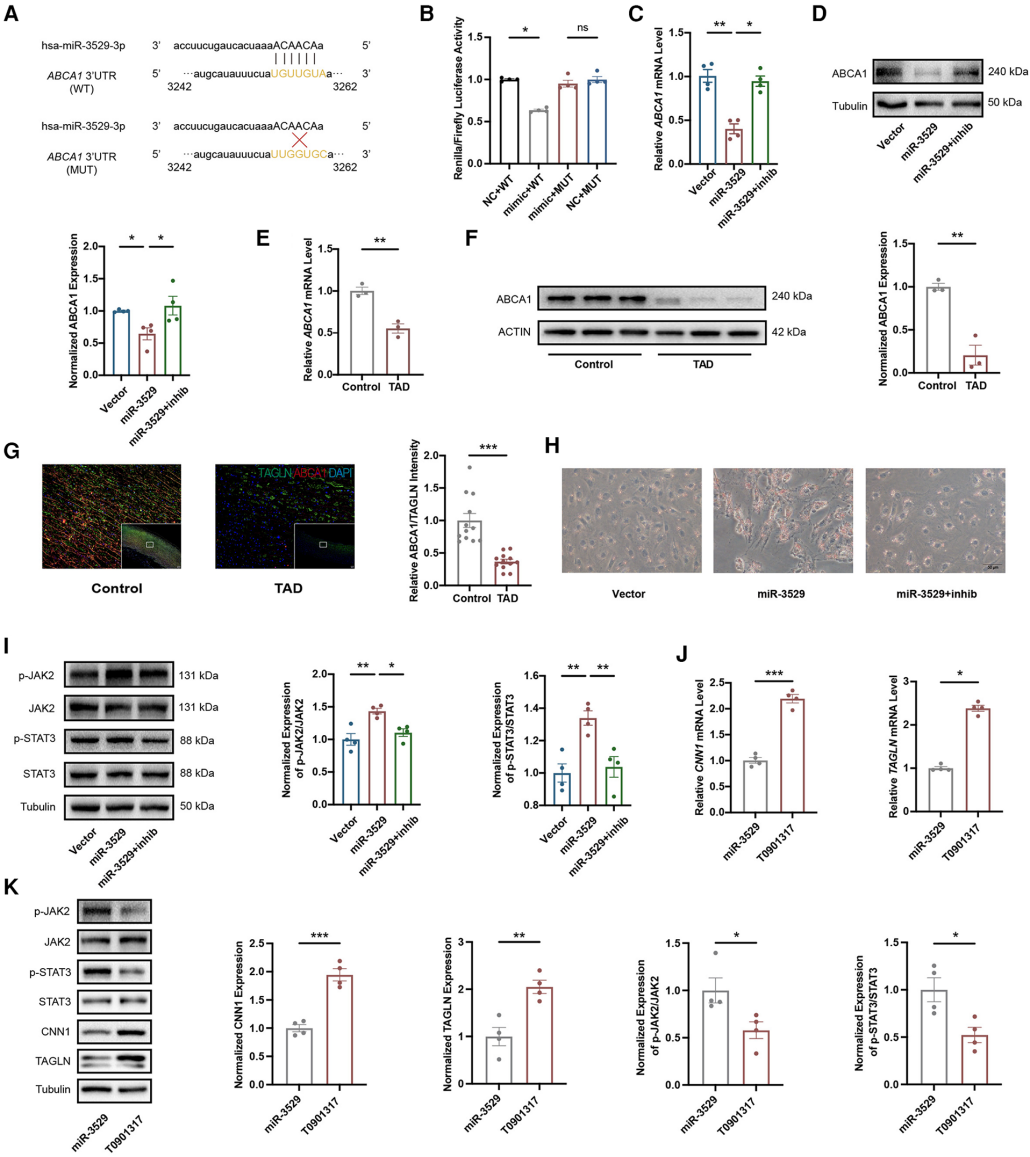
Identification of *ABCA1* as target gene of miR-3529 in HASMCs. **(A)** The theoretical miRNA-mRNA duplex between *ABCA1* and miR-3529. The pivotal binding sites (WT) and mutant sites (MUT) are highlighted with yellow fluorescence. **(B)** Relative luciferase activities (normalized to Firefly luciferase activities) of plasmids carrying *ABCA1* 3'UTR (WT)/MUT were examined in HEK-293 *T* cells with miR-3529 mimic or mimic NC (*n* = 4). **(C)** Expression of *ABCA1* in miR-3529-overexpressing HASMCs transfected with miR-3529 inhibitor (miR-3529 + inhib) or CTR inhibitor (miR-3529) compared to CTR cells transfected with CTR inhibitor (Vector), measured by qRT-PCR (*n* = 4). **(D)** Representative Western blots and quantification of ABCA1 protein expression in miR-3529 differently expressed HASMCs (*n* = 4). **(E)** Expression of *ABCA1* in TAD and control tissues, measured by qRT-PCR (*n* = 3). **(F)** Representative Western blots and quantification of ABCA1 protein expression in TAD and control tissues (*n* = 3). **(G)** Immunofluorescence staining for ABCA1 (red) and TAGLN (green), and quantification of relative ABCA1 intensity in TAD and control tissues (representative images chosen for similarity to the global quantification; scale bar, 200 µm; *n* = 12). **(H)** Representative Oil Red O-stained images of miR-3529 differently expressed HASMCs treated with Chol:MβCD, scale bar, 50 µm. **(I)** Representative Western blots and quantification of p-JAK2/p-STAT3 proteins expression in miR-3529 differently expressed HASMCs (*n* = 4). **(J)** Expression of HASMCs contractile markers in ABCA1-activated HASMCs vs. miR-3529-overexpressed HASMCs, measured by qRT-PCR (*n* = 4). **(K)** Representative Western blots and quantification of HASMCs contractile marker, p-JAK2/p-STAT3 proteins expression in ABCA1-activated HASMCs vs. miR-3529-overexpressed HASMCs (*n* = 4). Data are presented as means ± SEM. *P < 0.05, **P < 0.01, ***P < 0.001, ns, not significant.

A luciferase reporter assay was performed to further ascertain the direct effect of miR-3529 on *ABCA1*, in which binding of the miR-3529 seed sequence at the 3′UTR of *ABCA1* was monitored (Figure 7A). The luciferase activity of *ABCA1* 3′-UTR in the overexpression of the miR-3529-3p cell significantly reduces (about 0.7-fold) compared with that of scramble control, which verified that *ABCA1* is a direct target of miR-3529 (Figure 7B). Coherently, overexpression of miR-3529 induced the downregulation of ABCA1 both at mRNA and protein levels in HASMCs (Figures 7C,D). The expression of ABCA1 was also measured in vascular tissues. As expected, it was also significantly reduced in TAD tissues (Figures 7E–G). ABCA1 functions as a cholesterol efflux pump which is required for regulating reverse cholesterol transport (25, 26). After incubation with cholesterol for 48 h, HASMCs assumed the appearance of foam cells, with the majority of cells containing lipid droplets that are stained with Oil Red O, especially in miR-3529-overexpressing HASMCs (Figure 7H). These findings demonstrated that miR-3529 directly regulates ABCA1 expression. KEGG analysis of 77 DEGs identified by transcriptomics revealed enrichment of the JAK/STAT signaling pathway (Supplementary Figure S6). It is indicated that the JAK2/STAT3 signaling pathway serves as a downstream pathway of ABCA1 as well, regulating the expression of various inflammatory mediators (27). Hence, we detected the activation of JAK2/STAT3, and the results showed p-JAK2 and p-STAT3 was increased in miR-3529-overexpressing HASMCs (Figure 7I).

To further elucidate the role of the miR-3529/ABCA1 axis in HASMCs homeostasis, we reactivate ABCA1 expression by employing an ABCA1 agonist, T0901317, into miR-3529 overexpressed HASMCs. ABCA1 was significantly upregulated by treating with T0901317 for 12 h (Supplementary Figure S7). Meanwhile, the expression of contractile markers CNN1 and TAGLN are effectively upregulated in line with ABCA1 increasing, and concurrently inhibiting the activation of JAK2/STAT3 (Figures 7J,K). Therefore, miR-3529 intensifies disruption of homeostasis in HASMCs by activating JAK2/STAT3 signaling pathway via inhibition of ABCA1.

## 4 Discussion

TAD is a severe and life-threatening emergency without effective pharmacological therapy. Recently, many miRNAs including miR-21 and miR-134-5p have been identified as potential biomarkers and therapeutic targets for TAD (28, 29). In our present study, we screened out 14 up-regulated and 5 down-regulated miRNAs in TAD patients. Among them, miR-3529 expression was significantly elevated in vessel tissues from TAD (Figures 1E–H), which was determined in human aortic vessels for the first time. Further, its expression pattern in tissue and cells was determined from the miTED database (30). The data showed miR-3529 only expressed in human species among mammals, and rarely expressed in the aorta and SMC in normal conditions (Supplementary Tables S5, S6). This may be the reason why miR-3529-induced homeostasis disruption phenotypes were not altered obviously with inhibitor transfection in primary HASMCs (Supplementary Figures S5A–D). Meanwhile, its function in the pathological process of TAD remains explored.

VSMCs homeostasis disruption including phenotype switch is one major pathogenic mechanism of TAD by promoting adverse arterial wall remodeling (31, 32). With the upregulation of miR-3529, the homeostasis of VSMCs was disrupted, accompanied by the decrease of contractile markers TAGLN and CNN1, and induced a pro-inflammatory phenotype. The integrated-omics approach offers a comprehensive view of the whole transcriptome alteration and metabolic reprogramming signatures with miR-3529 overexpression, uncovering potential key target genes and pathways involved in the development of TAD. Here we first validated ABCA1 as a target of miR-3529 that disrupts HASMCs homeostasis.

There are several lines of evidence from clinical and preclinical studies that inflammation represents a critical response to vascular injury and is an essential component for TAD progression (33, 34). It is known that when ABCA1 is repressed in VSMCs, excess cholesterol accumulates contributing to foam cell formation and presenting inflammatory phenotype (35, 36). Given the interaction between cholesterol homeostasis and inflammation, miR-3529 may regulate HASMCs homeostasis by inhibiting ABCA1. This has led the research focus to shift towards looking at the anti-inflammatory benefits of ABCA1 in vascular disorder diseases. He et al. present evidence that partial or total ABCA1 deficiency is associated with a proinflammatory status in humans (37–39). Jiang et al. revealed the inflammation was facilitated by excess cholesterol accumulation in AD (40). In our study, total ABCA1 was diminished both at the mRNA and protein levels with miR-3529 abnormal expression in the medial layer of TAD patients (Figures 7E–G). JAK2/STAT3 is a classic inflammatory pathway, exacerbating vascular dysfunction and contributing to the progression of cardiovascular diseases (41, 42). As expected, the JAK2/STAT3 pathway was activated with overexpression of miR-3529, which disrupts HASMCs homeostasis. In line with these findings, miR-3529-overexpressing cells exhibited altered levels of IL6 and IFI44l, as inflammatory factors, may also be involved in the disruption of HASMCs homeostasis (Figure 6D). Nevertheless, these results implicated that ABCA1 is a potential anti-inflammatory target in HASMCs and led to increasing interest in investigating the benefit of ABCA1 in TAD.

In our study still has some limitations should be mentioned. Since miR-3529 is only expressed in homo sapiens in mammals, its function is hard to validate in animal models *in vivo*. Therefore, we only conducted validation on tissues from TAD patients and investigated the role of miR-3529 *in vitro*. Meanwhile, why miR-3529 is upregulated in TAD should be elucidated in the future.

In summary, we demonstrate the mechanism of miR-3529-induced VSMCs homeostasis disruption by integrative analysis of metabolomic and transcriptomic data. Another vital finding is miR-3529-induced regulation of ABCA1.miR-3529 plays a role in inducing lipid metabolism dysregulation and inflammatory responses through the ABCA1/JAK2/STAT3 pathway. A therapeutic application of inhibition miR-3529 or upregulation ABCA1 for TAD characterized by VSMCs homeostasis could be foreseen. Our study provides new and fundamental insights into the potential of miR-3529/ABCA1/JAK2/STAT3 against TAD.

## Data Availability

The raw data supporting the conclusions of this article have been deposited in China National Center for Bioinformation database (Accession Number: HRA007954, OMIX006829).
