# Supplementary figures and images for: miR-3529-3p/ABCA1 axis regulates smooth muscle cell homeostasis by enhancing inflammation via JAK2/STAT3 pathway
Source: Front Cardiovasc Med. 2024 Aug 27;11:1441123. doi: 10.3389/fcvm.2024.1441123 (PMC11384995; doi:10.3389/fcvm.2024.1441123)

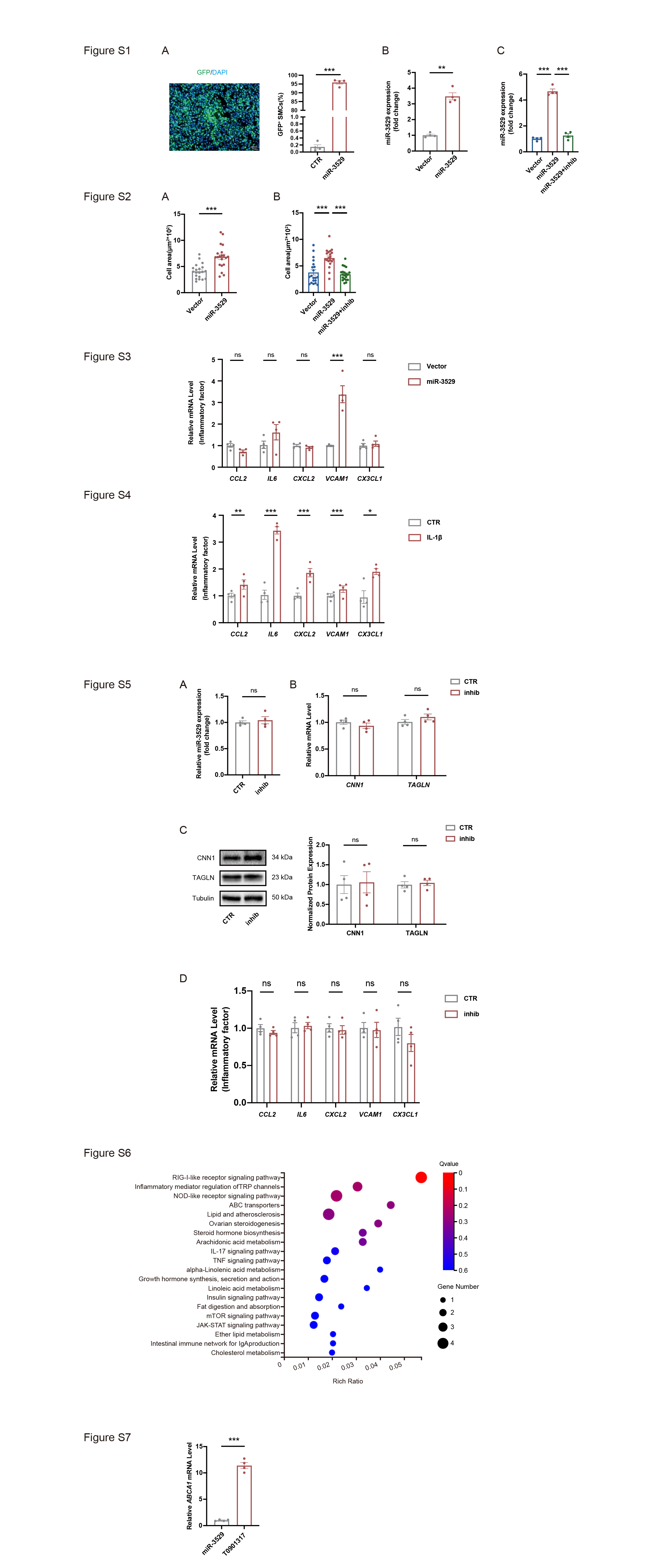

Supplement: Supplementary file 1 [file Image1.jpeg]
