# Supplementary material for: miR-3529-3p/ABCA1 axis regulates smooth muscle cell homeostasis by enhancing inflammation via JAK2/STAT3 pathway
Source: Front Cardiovasc Med. 2024 Aug 27;11:1441123. doi: 10.3389/fcvm.2024.1441123 (PMC11384995; doi:10.3389/fcvm.2024.1441123)
Supplement: Supplementary file 2 [file Datasheet1.docx]

**Table S1.** **The fragment of miR-3529 overexpression sequence**

| **Forward** | **Reverse** |
| --- | --- |
| CTAGAAACAACAAAATCACTAGTCTTCCATTCAAGAGATGGAAGACTAGTGATTTTGTTGTTTTTTTG | AATTCAAAAAAACAACAAAATCACTAGTCTTCCATCTCTTGAATGGAAGACTAGTGATTTTGTTGTTT |

The oligo DNA sequence of mature miR-3529-3p was labeled with fluorescent yellow; cloning XbaⅠ and EcoRⅠ sites are underlined.

**Table S2. Primers used in this study for qRT-PCR experiments**

| Genes | Primers | |
| --- | --- | --- |
|  | Forward | Reverse |
| TAGLN | AGTGCAGTCCAAAATCGAGAAG | CTTGCTCAGAATCACGCCAT |
| CNN1 | AACCATACACAGGTGCAGTC | GATGTTCCGCCCTTCTCTTAG |
| CCL2 | AAGCTCGCACTCTCGCCTCCA | GCATTGATTGCATCTGGCTGAGCG |
| IL6 | AAATTCGGTACATCCTCGACGG | GGAAGGTTCAGGTTGTTTTCTGC |
| CXCL2 | GCAGGGAATTCACCTCAAGA | AGCTTCCTCCTTCCTTCTGG |
| VCAM1 | CAGTAAGGCAGGCTGTAAAAGA | TGGAGCTGGTAGACCCTCG |
| CX3CL1 | ACCACGGTGTGACGAAATG | TGTTGATAGTGGATGAGCAAAGC |
| GLUT1 | CTGCTCATCAACCGCAAC | CTTCTTCTCCCGCATCATCT |
| HK2 | GAGCCATCCTGCAACACTTAGG | CAGTGCACACCTCCTTAACAATG |
| LDHA | AGCCCGATTCCGTTACCT | CACCAGCAACATTCATTCCA |
| PDK1 | TATGGATTGCCCATATCACG | CATCTGTCCCGTAACCCTCT |
| ABCA1 | AGTACCCCAGCCTGGAACTT | TGGGTTTCCTTCCATACAGCG |
| 18s | GTAACCCGTTGAACCCCATT | CCATCCAATCGGTAGTAGCG |

**Table S3. List of primary antibodies**

| Antibodies | SOURCE | IDENTIFIER |
| --- | --- | --- |
| TAGLN | Abcam | Cat# ab14106; RRID: AB_443021 |
| CNN1 | Abcam | Cat# ab46794; RRID: AB_2291941 |
| ABCA1 | Abcam | Cat# ab125064; RRID: AB_10973184 |
| Tubulin | Zenbio | Cat# 380628 |
| TAGLN | Proteintech | Cat# 60213-1-Ig; RRID: AB_11043177 |
| ABCA1 | Proteintech | Cat# 26564-1-AP; RRID: AB_3085884 |
| Phospho-JAK2 | Zenbio | Cat# R381556 |
| JAK2 | Zenbio | Cat# R24775 |
| Phospho-STAT3 | Zenbio | Cat# R381552 |
| STAT3 | Zenbio | Cat# R22785 |
| 𝛃-Actin | Abcam | Cat# ab8226; RRID: AB_306371 |

**Table S4. Expression profiles (TPM) of 19 miRNAs in TAD and control tissues**

| Gene ID | control-1 Expression | control-2 Expression | control-3 Expression | TAD-1 Expression | TAD-2 Expression | TAD-3 Expression |
| --- | --- | --- | --- | --- | --- | --- |
| hsa-let-7c-3p | 5.917 | 2.365 | 2.516 | 0.5 | 0.183 | 0.555 |
| hsa-miR-10a-3p | 2.004 | 3.118 | 0.694 | 0 | 0.091 | 0.278 |
| hsa-miR-10a-5p | 3319.672 | 3394.022 | 2024.877 | 66.105 | 612.503 | 532.848 |
| hsa-miR-128-2-5p | 0 | 0 | 0.087 | 0.428 | 0.365 | 0.069 |
| hsa-miR-1307-5p | 35.499 | 16.128 | 0.868 | 0.5 | 0.821 | 0.278 |
| hsa-miR-135a-5p | 13.646 | 46.342 | 19.175 | 0.999 | 4.289 | 2.568 |
| hsa-miR-155-5p | 174.825 | 89.78 | 280.517 | 328.313 | 300.594 | 549.3 |
| hsa-miR-203a-3p | 5.63 | 6.344 | 3.644 | 0.357 | 0.639 | 1.319 |
| hsa-miR-205-5p | 14.791 | 0.86 | 0 | 0 | 0.274 | 0.069 |
| hsa-miR-335-3p | 307.566 | 146.659 | 90.411 | 2.784 | 8.487 | 10.899 |
| hsa-miR-3529-3p | 2.099 | 0.538 | 5.119 | 303.756 | 174.023 | 341.187 |
| hsa-miR-378d | 3.722 | 6.129 | 2.082 | 0.071 | 0.091 | 0 |
| hsa-miR-431-5p | 6.203 | 3.011 | 4.078 | 12.921 | 3.559 | 6.248 |
| hsa-miR-4454 | 2.672 | 1.935 | 1.562 | 33.552 | 12.867 | 8.261 |
| hsa-miR-490-5p | 6.585 | 7.419 | 2.603 | 0 | 0 | 0 |
| hsa-miR-509-3p | 16.509 | 0.968 | 2.343 | 0.143 | 0 | 0.069 |
| hsa-miR-551b-3p | 3.913 | 4.516 | 11.887 | 0.286 | 0.456 | 0.069 |
| hsa-miR-99a-3p | 17.941 | 22.257 | 8.763 | 3.498 | 3.559 | 3.957 |
| novel-hsa-miR63-5p | 13.455 | 6.666 | 3.644 | 1.927 | 1.278 | 1.944 |

**Table S5. Expression** **(RPM) of miR-3529 in normal tissues**

| Ranking | tissue | expression |
| --- | --- | --- |
| 1 | Brain stem | 0.31 |
| 2 | Renal tubular cells | 0.25 |
| 3 | Hard palate | 0.185 |
| 4 | Testis | 0.14993151 |
| 5 | Cartilage | 0.12857143 |
| 6 | Connective, subcutaneous and other soft tissues of head, face, and neck | 0.122 |
| 7 | Neuron | 0.095 |
| 8 | Adipose-derived stem cells | 0.095 |
| 9 | Peripheral nerves and autonomic nervous system of upper limb and shoulder | 0.09 |
| 10 | Head, face or neck | 0.07 |
| 11 | Connective, subcutaneous and other soft tissues of thorax | 0.065 |
| 12 | Neural stem cells | 0.035 |
| 13 | Myometrium | 0.03 |
| 14 | Brain | 0.02898765 |
| 15 | Mediastinum | 0.024 |
| 16 | Corpus uteri | 0.02341463 |
| 17 | Uterus | 0.0182 |
| 18 | Frontal lobe | 0.016 |
| 19 | Descending colon | 0.01277778 |
| 20 | Lower third of esophagus | 0.01119403 |
| ··· | ··· | ··· |
| 151 | Aorta | 0 |
| ··· | ··· | ··· |

**Table S6. Expression (RPM) of miR-3529 in normal cells**

| Ranking | cell | expression |
| --- | --- | --- |
| 1 | SC14-069 | 1.77 |
| 2 | SC13-044 | 1.3 |
| 3 | SC12-022 | 0.995 |
| 4 | SC12-024 | 0.595 |
| 5 | SC12-025 | 0.555 |
| 6 | Huh7 | 0.35666667 |
| 7 | SC11-016 | 0.3 |
| 8 | SC14-066 | 0.295 |
| 9 | SC11-015 | 0.28666667 |
| 10 | SC13-043 | 0.268 |
| 11 | SC14-067 | 0.25666667 |
| 12 | SC11-013 | 0.245 |
| 13 | HBEC1MBT | 0.155 |
| 14 | C666 | 0.14 |
| 15 | HOP-62 | 0.125 |
| 16 | SC13-049 | 0.11666667 |
| 17 | C8166 | 0.1 |
| 18 | SW527 | 0.1 |
| 19 | HCT-15 | 0.09 |
| 20 | TZM-bl | 0.08 |
| ··· | ··· | ··· |
| 152 | PrSMC | 0 |
| ··· | ··· | ··· |
