# Supplementary material for: miR-3529-3p/ABCA1 axis regulates smooth muscle cell homeostasis by enhancing inflammation via JAK2/STAT3 pathway
Source: Front Cardiovasc Med. 2024 Aug 27;11:1441123. doi: 10.3389/fcvm.2024.1441123 (PMC11384995; doi:10.3389/fcvm.2024.1441123)
Supplement: Supplementary file 3 [file Datasheet2.docx]

**Figure S1. miR-3529 expression and modulation in HASMCs.** (A) Representative images and quantification of GFP in miR-3529-overexpressing HASMCs (n=4). (B) qRT-PCR detected the expression of miR-3529 in HASMCs vs. CTR cells (n=4). (C) qRT-PCR detected the expression of miR-3529 in miR-3529 differently expressed HASMCs, measured by qRT-PCR (n=4). Data are presented as means ± SEM. **P < 0.01, ***P < 0.001.

**Figure S2. The cell size of HASMCs with different miR-3529-3p expressions.** (A) Relative quantification of cell size in miR-3529-overexpressing HASMCs vs. CTR cells (n=20). (B) Relative quantification of cell size in miR-3529 differently expressed HASMCs (n=20). Data are presented as means ± SEM. ***P < 0.001.

**Figure S3.** Expression of HASMCs pro-inflammatory genes in miR-3529-overexpressing HASMCs vs. CTR cells, measured by qRT-PCR (n=4). Data are presented as means ± SEM. *** P < 0.001, ns, not significant.

**Figure S4.** Expression of HASMCs pro-inflammatory genes in HASMCs treated or untreated with IL-1β (20 ng/mL, 24 h), measured by qRT-PCR (n=4). Data are presented as means ± SEM. *P < 0.05, **P < 0.01, ***P < 0.001.

**Figure S5. miR-3529 inhibitor impact on HASMCs homeostasis.** (A) Expression of miR-3529 in HASMCs transfected with miR-3529 inhibitor (inhib) or CTR inhibitor (CTR), measured by qRT-PCR (n=4). (B) Expression of HASMCs contractile markers, measured by qRT-PCR (n=4). (C) Representative Western blots and quantification of HASMCs contractile marker proteins expression (n=4). (D) Expression of HASMCs pro-inflammatory genes in HASMCs transfected with miR-3529 inhibitor (inhib) or CTR inhibitor (CTR) treated with IL-1β (20 ng/mL,24 h), measured by qRT-PCR (n=4). Data are presented as means ± SEM. ns, not significant.

**Figure S6.** **KEGG pathway analysis (signaling pathways) of 77 DEGs.**

**Figure S7.** Expression of ABCA1 in ABCA1-activating HASMCs vs. miR-3529-overexpressed HASMCs, measured by qRT-PCR (n=4). Data are presented as means ± SEM. ***P < 0.001.
